# Serotonin and Dopamine Protect from Hypothermia/Rewarming Damage through the CBS/ H_2_S Pathway

**DOI:** 10.1371/journal.pone.0022568

**Published:** 2011-07-27

**Authors:** Fatemeh Talaei, Hjalmar R. Bouma, Adrianus C. Van der Graaf, Arjen M. Strijkstra, Martina Schmidt, Robert H. Henning

**Affiliations:** 1 Department of Clinical Pharmacology (FB20), University Medical Center Groningen, University of Groningen, Groningen, The Netherlands; 2 Angteq B.V., Turftorenstraat, Groningen, The Netherlands; 3 Department of Chronobiology, University of Groningen, Groningen, The Netherlands; 4 Department of Molecular Pharmacology, Faculty of Pharmacy, University of Groningen, Groningen, The Netherlands; Universidade Federal do Rio de Janeiro, Brazil

## Abstract

Biogenic amines have been demonstrated to protect cells from apoptotic cell death. Herein we show for the first time that serotonin and dopamine increase H_2_S production by the endogenous enzyme cystathionine-β-synthase (CBS) and protect cells against hypothermia/rewarming induced reactive oxygen species (ROS) formation and apoptosis. Treatment with both compounds doubled CBS expression through mammalian target of rapamycin (mTOR) and increased H_2_S production in cultured rat smooth muscle cells. In addition, serotonin and dopamine treatment significantly reduced ROS formation. The beneficial effect of both compounds was minimized by inhibition of their re-uptake and by pharmacological inhibition of CBS or its down-regulation by siRNA. Exogenous administration of H_2_S and activation of CBS by Prydoxal 5′-phosphate also protected cells from hypothermic damage. Finally, serotonin and dopamine pretreatment of rat lung, kidney, liver and heart prior to 24 h of hypothermia at 3°C followed by 30 min of rewarming at 37°C upregulated the expression of CBS, strongly reduced caspase activity and maintained the physiological pH compared to untreated tissues. Thus, dopamine and serotonin protect cells against hypothermia/rewarming induced damage by increasing H_2_S production mediated through CBS. Our data identify a novel molecular link between biogenic amines and the H_2_S pathway, which may profoundly affect our understanding of the biological effects of monoamine neurotransmitters.

## Introduction

Ischemia is a condition suffered by cells in tissues when deprived of blood flow due to inadequate nutrient and oxygen supplementation. The restoration of blood flow following an ischemic condition causes reperfusion damage [1] mainly due to the rapid generation of ROS from the start of reperfusion [2] and characterized by apoptotic cell death [3]. Likewise, many mammalian cell types are vulnerable to prolonged and profound hypothermic storage mainly due to the burst of reactive oxygen species (ROS). Particularly during the rewarming phase, low ATP production, Ca^2+^ overload and cell swelling result in apoptotic cell death [4], [5]. Thus, the apoptotic damage brought about by either ischemia or hypothermia results from a burst in ROS formation during reperfusion or rewarming. Several observations suggest that catecholamines protect from cell death after hypothermia and the subsequent rewarming. Dopamine has been shown to limit oxidative stress in cultured cells during cold storage [6] and to improve kidney graft function after transplantation [7]. In initial experiments in search of mechanisms conveying a natural resistance to hypothermia on cells of a hibernating species, the Syrian hamster, we found that their ductus deferens (DDT-1 MF2) cells are protected from hypothermia induced apoptosis. This was found to be due to the secretion and reuptake of serotonin, a tryptamine (non-catecholamine) bioamine, by these cells and conveyed by increasing the production of endogenous H_2_S.

Cystathionine-β-synthase (CBS) is the most likely endogenous candidate enzyme to increase H_2_S production. Endogenous H_2_S is mainly synthesized by CBS and cystathionine-γ-lyase [8], [9]. Both enzymes depend on pyridoxal 5′-phosphate (PLP) as a cofactor [10]. However, only CBS contains a heme moiety, which may bind oxygen and make the enzyme function dependent on oxygen levels, as demonstrated in recombinant human CBS [11]. In addition, a range of biogenic amines, including serotonin, dopamine and noradrenalin bind the heme moiety of various enzymes, possibly modulating different cell functions [12]. Therefore, in this study we examined the involvement of CBS and H_2_S production in the protective effect of serotonin and dopamine on cold induced cellular damage in a cell line that showed the highest vulnerability to hypothermia, by studying cell numbers, caspase activity, and ROS formation. Moreover, we examined the expression of CBS in serotonin and dopamine treated rat tissues after static cold preservation in parallel to apoptosis and tissue acidosis/ischemia.

## Methods

### Cell culture and hypothermic insult

Five cell lines including NRK-52E (normal rat kidney cells, ATCC, USA; 87012902), DDT-1 MF2 (hamster ductus deferens muscle cells, ATCC, USA CRL1701) and A7R5 (rat vascular smooth muscle cells, ATCC, USA CRL1444) cultured in DMEM (Gibco) and SMAC (rat smooth muscle aortic cells, ATCC, USA CRL1476) and THMC (transformed human mesangial cell IP15) cultured in DMEM/F12 (Gibco) were chosen to study hypothermia resistance. All media were supplied with 10% (v/v %) fetal calf serum and 100 U/mL penicillin, 100 µg/mL streptomycin and cultured at 37°C in 5% CO_2_ in 25 cm^2^ or 75 cm^2^ flasks. Cells were plated in 6 or 96 wells plates and grown to confluence. Induction of cellular damage by hypothermia consisted of placing cells at 3°C for 24 h. Cell viability was measured by MTS assay (Promega) according to the manufacturer's instructions. For the latter, 20 µl of MTS solution was added to each well and cells were subsequently placed in the incubator at 37°C in 5% CO_2_ for 3 h after which cell viability was determined by measuring absorption at 490 nm.

### Production of H_2_S

H_2_S was assayed according to Stipanuk and Beck [13] and Zhao et al. [14] with some modifications. Zinc Acetate (1%) was added to each 4 ml of cell free supernatant to trap the evolved H_2_S. Diamine-ferric solution was prepared by mixing 100 µl of a 400 mg N, N-dimethyl-p-phenylenediamine dihydrochloride dissolved in 10 ml 6 M HCl and 100 µl of 600 mg ferric chloride in 10 ml 6 M HCl. Two hundred µl of this mixture was added to the cell supernatant and after an incubation time of 30 min at 37°C, the amount of methylene blue formed in the supernatant was measured at a wavelength of 670 nm. Blanks were made following the same procedure without cells. The concentration of H_2_S was calculated by extrapolation using a standard curve obtained from different concentrations of methylene blue and spectrophotometric measurement at a wavelength of 670 nm.

### Hypothermia challenge and MTS assay

Confluent SMAC cells in 96 wells were treated with serotonin (30 µM), dopamine (20 µM) and pyridoxal 5′-phosphate (PLP, 50 µM). After 15 min the plates were placed at 3°C for 24 hr. Incubation of SMAC with NaHS/sodium hydrosulfate (0.2 mM) was performed just before rewarming cells, to assure presence of H_2_S during the rewarming phase, as NaHS only briefly releases H_2_S after being dissolved. Non-treated cells were kept as controls. MTS assay was performed 15 min after rewarming as previously described.

### siRNA for cystathionine-β-synthase

The expression of CBS in SMAC was reduced by applying a predesigned siRNA (sc-60336, Santa Cruz) and compared to a silencer negative control (Ambion, AM4644). DDT-1 and SMAC cells at 60–80% confluence were seeded in 96 or 6 well plates in antibiotic-free normal growth medium supplemented with FCS. Cells were transfected using lipofectamine 2000 (Invitrogen) at a final concentration of 100 pmol siRNA in 5 µl lipofectamine for each well in a 6 well plate and 5 pmol siRNA in 0.25 µl lipofectamine for each well in a 96 well plate. After 24 h, the medium was changed to the medium containing antibiotics and FCS. Cells were left to proliferate for 48 hr at 37°C. Then, control cells, siRNA treated cells and cells transfected with the negative control silencer were incubated at 37°C or 3°C in the presence and absence of serotonin (30 µM) or dopamine (20 µM) for 24 h.

### Measurement of reactive oxygen species

Reactive oxygens species (ROS) were detected as described in the supplementary information using the fluorescent probe CM-H2-DCFDA (2,7 dichloroflourescein diacetate), which detects both formation of superoxide anions and hydroxyl radicals.

### Experiments on tissue and analysis

Tissue samples (lung, kidney, liver and heart) from male Sprague Dawley rats (300–350 g) were harvested and each cut into three separate pieces and placed in glass containers containing 2 ml of PBS (phosphate buffered saline, pH 7.4) either treated with serotonin (90 µM) or dopamine (60 µM) for 30 min at 37°C prior to 24 h of hypothermic treatment (3°C) followed by 30 min of rewarming at 37°C. The same procedure was followed for control samples using untreated PBS. As tissue pH monitoring has proven to be a valuable means in assessing tissue ischemia [15], pH of each medium was assessed after rewarming. Tissue slices for immunohistological studies were placed in zinc fixative solution (0.1 M Tris-HCl, pH 7.4; 0.05% calcium acetate, 0.5% zinc acetate, 0.5% zinc chloride) at room temperature for 12 h and then processed and embedded in paraffin. Paraffin blocks were cut in 3 µm sections, deparaffinized, and submitted to CBS antibody staining according to the procedure described in Histology and Immunostaining in supplementary information. Further, the apoptosis level in tissue slices was also investigated by measuring caspase activity, following the procedure described in supplementary information. Animal experiments were approved by the Ethics committee of the university medical center Groningen (DEC 5920).

### Statistical analysis

Statistical data analyses were performed using the One-way ANOVA with Tukey's test (GraphPad Prism version 5) and p<0.05 was considered as statistically significant.

* Details on the experiments are included as supplemental information with the article.

## Results

### Resistance to hypothermic cell injury depends on cellular uptake of serotonin and dopamine

**Figure 1:**
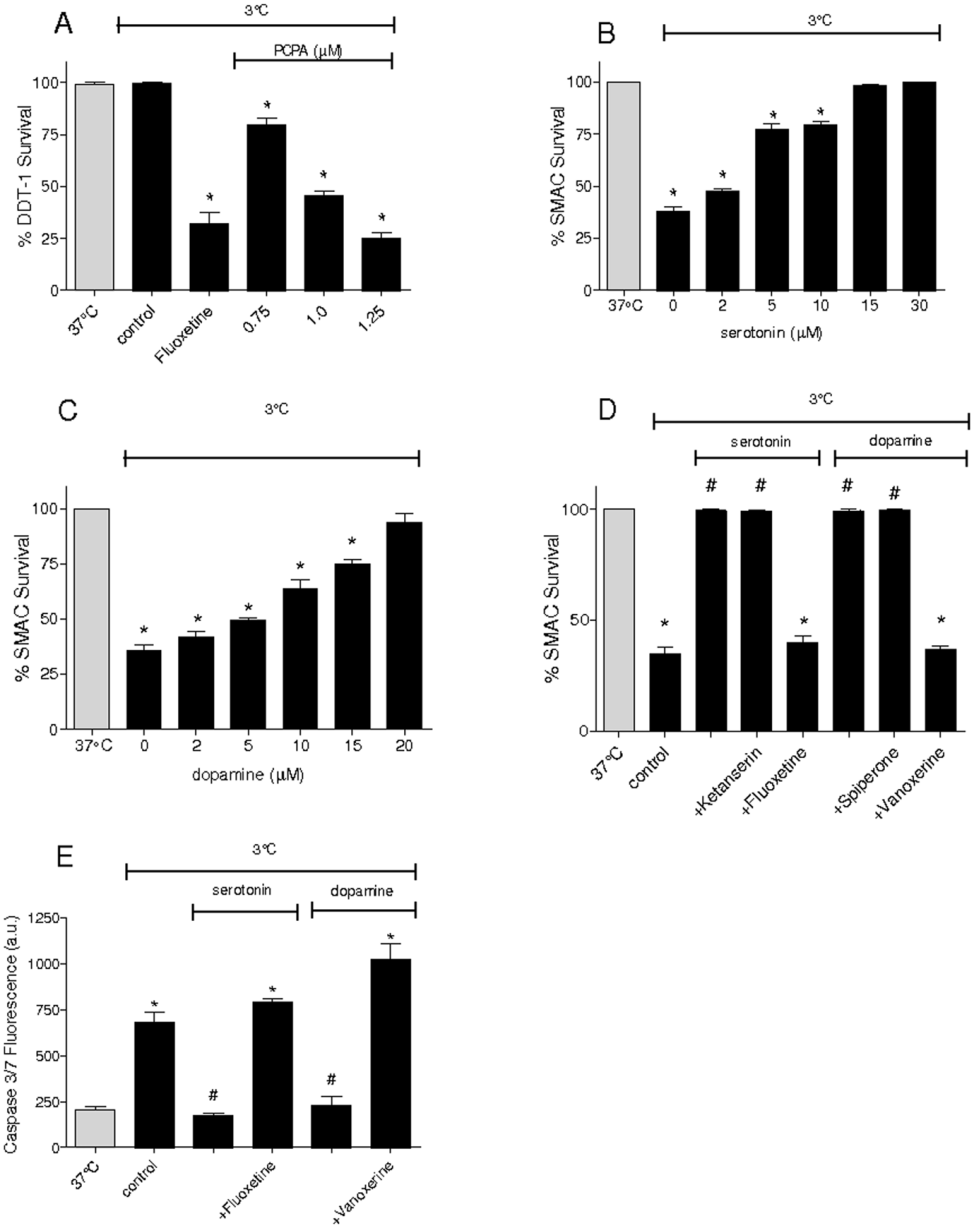
Serotonin and dopamine protect cells from hypothermia/rewarming cell death through an intracellular action. Cells subjected to hypothermia (black bars) were incubated at 3°C for 24 h, followed by rewarming to 37°C for 3 h, and compared to non-cooled control cells (37°C, gray bars). Cell survival was assessed by adding MTS to the cells upon rewarming and spectrophotometrical formazon measurement. (**A**) DDT-1 cells show natural resistance to hypothermia, which is abrogated by the serotonin transporter (SERT) inhibitor fluoxetine (Fluox, 1 µM, 15 min) and pretreatment with the tryptophan hydroxylase inhibitor parachlorophenylalanine (PCPA, 24 h). (**B to C**) Concentration-dependent inhibition of hypothermic cells death by serotonin (B) and dopamine (C) in SMAC. (**D**) The protective effect of serotonin (30 µM, 15 min) and dopamine (20 µM, 15 min) pretreatment on hypothermic cell death is precluded by inhibition of their respective transporters with fluoxetine (1 µM, 15 min) and vanoxerine (1 µM, 15 min), but unaffected by non-specific receptor antagonists ketanserin (1 µM, 15 min) and spiperone (1 µM, 15 min). (**E**) Serotonin (30 µM, 15 min) and dopamine (20 µM, 15 min) pretreatment prevent caspase3/7 activation induced by hypothermia in SMAC cells, which is precluded by inhibition of their uptake by fluoxetine (1 µM, 15 min) and vanoxerine (1 µM, 15 min). ANOVA tests, different from non-cooled cells (37°C) P<0.05 (*); different from untreated hypothermic cells (Con) P<0.05 (#). Experiments consist of n≥4. Means ± SEM.

Survival of DDT-1 MF2 cells (DDT-1 cells) was unaffected by hypothermic storage (3°C, 24 h) and subsequent rewarming (37°C, 3 h), whereas other cell lines showed substantial cell death (Figure S1A). Medium obtained from hypothermic DDT-1 cells (3°C, 18 hrs) protected vulnerable cell lines against hypothermic injury, whereas medium from normothermic DDT-1 cells was ineffective (Figure S1B and Text S1; Supplemental Information Materials and Methods). Thus, hypothermia induced the release of a protective factor from DDT-1 cells into the medium. Staining of DDT-1 cells with Ehrlich reagent or serotonin antibody demonstrated the presence of serotonin containing vesicles in DDT-1 cells (Figure S2). Mass spectrometry confirmed the released compound to be serotonin (medium concentration at 37°C: <3.0 µM; at 37°C with 1 µM fluoxetine: 20.5 µM; at 3°C: 24.9 µM). To demonstrate that serotonin conveys resistance to hypothermic cell death in DDT-1, synthesis of serotonin, its intracellular uptake and its receptors were inhibited by pharmacological interventions. Four days of pretreatment with the tryptophan hydroxylase inhibitor parachlorophenylalanine (PCPA, 1 µM) decreased intracellular serotonin content of DDT-1 cells by 50±10% (n = 8) and concentration-dependently decreased its survival to subsequent hypothermia (24 hr, 3°C; figure 1A). Blockade of serotonin uptake in DDT-1 by inhibition of its transporter (SERT) with fluoxetine (1 µM) abrogated the natural resistance of DDT-1 to hypothermic cell death (24 h, 3°C) and resulted in cell death of over 50% (Figure 1A). In contrast, blockade of serotonin receptors with ketanserin (1 µM) did not affect DDT-1 cell survival following hypothermic treatment (24 h, 3°C; data not shown).

These experiments identify the monoamine serotonin (5-hydroxytryptamine) as a protective compound against hypothermic cell death. Previously, dopamine (hydroxytyramine) was found to exert similar effects in cultured endothelial cells [6]. Thus, the actions of both compounds were explored in rat smooth muscle aortic cells (SMAC), shown to be vulnerable to hypothermic cell death (Figure S1). Pretreatment (15 min, 37°C) of SMAC with either serotonin or dopamine provided a concentration-dependent resistance to hypothermic cell death (24 h, 3°C; figure 1B and 1C). Inhibition of the serotonin transporter SERT (fluoxetine, 1 µM) and dopamine transporter DAT (vanoxerine, 1 µM), however, completely abrogated serotonin and dopamine induced resistance to hypothermic damage in SMAC (Figure 1D). In contrast, non-specific antagonists of serotonin (ketanserin, 1 µM) and dopamine (spiperone, 1 µM) receptors did not affect serotonin or dopamine induced protection of SMAC from hypothermic cell death (Figure 1D). Serotonin or dopamine also prevented hypothermia/rewarming induced increase of caspase 3/7 activity, which was abrogated by inhibitors of re-uptake (Figure 1E). Together, these experiments identify serotonin to protect from cold-induced cell death and demonstrate that the protective effect of serotonin and dopamine depends on their cellular uptake and is independent of the presence of either receptor.

### Serotonin and dopamine induce CBS mediated H_2_S production

**Figure 2:**
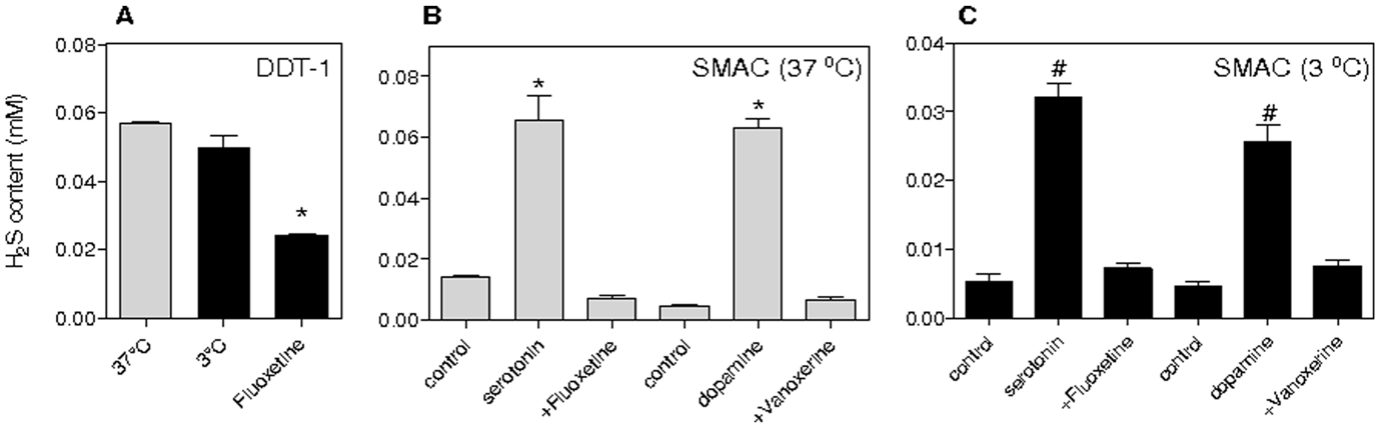
Induction of cellular H_2_S production by serotonin and dopamine. H_2_S content was measured in cell medium of non-cooled cells (gray bars, 37°C) and hypothermic cells (black bars, 3°C) after incubation for 24 h. (**A**) H_2_S content in DDT cells was unaffected by cooling, but reduced by the serotonin transporter (SERT) inhibitor fluoxetine (1 µM, 15 min pretreatment prior to cooling). (**B** to **C**) Serotonin (30 µM) and dopamine (20 µM) induce H_2_S production in SMAC both at 37°C and 3°C compared o untreated cells (control), which is blocked by inhibition of their respective transporters fluoxetine (1 µM, 15 min) and vanoxerine (1 µM, 15 min). ANOVA tests, different from non-cooled cells (37°C or control) P<0.05 (*); different from untreated hypothermic cells (Con) P<0.05 (#). Experiments consist of n≥4. Means ± SEM.

As medium from hypothermic DDT-1 cells slightly smelled like certain sulfur-containing compounds, the cellular production of H_2_S was investigated. Therefore, H_2_S content in homogenates and medium of DDT-1 and SMAC was measured. While DDT-1 cells produce considerable amounts of H_2_S at 37°C (Figure 2A), SMAC show only a marginal production (Figure 2B). H_2_S production of DDT-1 was reduced by inhibition of SERT (fluoxetine 1 µM, Figure 2A). Incubation of SMAC at 37°C with serotonin and dopamine increased H_2_S production (Figure 2B). Pretreatment of SMAC with serotonin and dopamine also strongly increased H_2_S production during a subsequent hypothermic treatment (3°C, 24 h), which was abrogated by co-treatment with their respective uptake inhibitors (Figure 2C).

**Figure 3:**
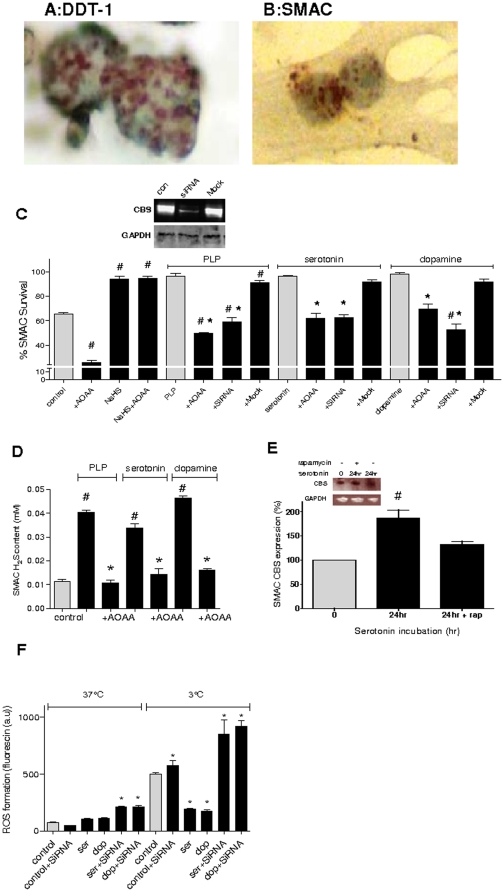
Serotonin and dopamine prevent hypotherma/rewarming cell death via increased H_2_S production through upregulation and allosteric activation of cystathionine-β-synthase (CBS). (**A** and **B**) Immunostaining demonstrating the expression of CBS in DDT-1 (A) and SMAC (B). Magnification 80×. (**C**) Downregulation of CBS by siRNA precludes protection of SMAC from hypothermic cell death by serotonin (30 µM), dopamine (20 µM) and PLP (50 µM). NaHS protects cells against hypothermia even in the presence of AOAA (1 mM) an inhibitor of CBS. The hypothermia protocol consisted of 24 h at 3°C for, followed by rewarming to 37°C for 3 h. Cell survival was assessed by adding MTS to the cells upon rewarming and spectrophotometrical formazon measurement. Inset: Silencing RNA substantially decreases the expression of CBS in SMAC cells. con: untreated cells, mock: negative control siRNA. (**D**) The increase in production of H_2_S as measured in cell medium of hypothermic cells (24 h at 3°C) treated with serotonin (30 µM) or dopamine (20 µM) was abrogated by pretreatment of the cells with the inhibitor of CBS, amino-oxyacetic acid (+AOAA, 1 mM, 15 min at 37°C+24 hr at 3°C ). (**E**) Treatment with serotonin (30 µM, 15 min at 37°C+24 hr at 3°C )) upregulates CBS expression, which is prevented by pretreatment with rapamycin (rap, 30 nM, 15 min at 37°C+24 hr at 3°C). Inset: western blot with time points as indicated. (**F**) Cooling SMAC induces the production of ROS in cells which is reduced by dopamine and serotonin treatment and aggravated by CBS siRNA transfection. ROS formation is measured by the level of Fluorescin fluorescence in SMAC in dopamine and serotonin treated cells compared to controls at 3°C and 37°C individually. Experiments consist of n≥3. Means ± SEM. # indicates significant difference to untreated control and * indicates significant difference to control within each treatment group.

Immunohistological staining established the expression of CBS both in DDT-1 and SMAC (Figure 3A and 3B). To confirm that the protective effect was due to CBS mediated production of H_2_S, the expression of the enzyme was reduced using siRNA in SMAC prior to stimulation with serotonin, dopamine and the endogenous activator of CBS, PLP. Silencing RNA substantially reduced CBS expression compared to control (Figure 3C, inset). Importantly, downregulation of CBS expression with siRNA inhibited H_2_S production and attenuated the serotonin, dopamine and PLP induced resistance to hypothermic cell death in SMAC (Figure 3C). In addition, pharmacological blockade of CBS by amino-oxyacetic acid (AOAA, 1 mM) also diminished the protective effect of serotonin, dopamine and PLP on hypothermic cell death (24 h, 3°C; figure 3C). Finally, NaHS, as a substance which releases H_2_S, was used as a second control to demonstrate the protective effect of H_2_S against hypothermia induced cell damage. Pretreatment with NaHS (0.2 mM) protected against hypothermia/rewarming even in the presence of AOAA (Figure 3C). PLP, serotonin and dopamine increase H_2_S production in SMAC while addition of AOAA to each treatment does not affect the level compared to control (Figure 3D). Collectively, these data show that the protective effect of serotonin and dopamine against hypothermic cell death in SMAC is dependent on CBS mediated H_2_S production.

### Serotonin and dopamine upregulate and activate cystathionine-β-synthase and inhibit hypothermia/rewarming induced ROS formation in CBS containing cells

Next, we examined the effect of serotonin and dopamine on the expression and the activity of CBS. Pretreatment of SMAC with serotonin and dopamine upregulated the expression of CBS, an effect being most pronounced after 24 hr of incubation (Figures 3E and S3A). Upregulation was likely dependent on the increase in protein synthesis of CBS and it was attenuated by rapamycin (30 nM), an inhibitor of protein synthesis by inhibition of mammalian target of rapamycin (mTOR) [16]. In addition to upregulation, we examined whether increased H_2_S production was due to activation of CBS [17], [18]. To substantiate allosteric activation of CBS by serotonin and dopamine, their action on isolated CBS was examined in an *in vitro* assay, employing PLP as a positive control. In this assay at 37°C, serotonin and dopamine substantially induced the formation of H_2_S, as was observed with PLP. In addition, both compounds increased H_2_S production of isolated CBS (Figure S3B and Text S1). Thus, the area under the curve (AUC) of H_2_S production following 10 min incubation increased significantly from 13.9±4.4 in controls to 56.3±13.7, 111.3±16.4 and 47.9±3.6 after stimulation with PLP, serotonin and dopamine, respectively (all p<0.05 compared to control). These results implicate that serotonin and dopamine activate CBS allosterically. Collectively, these data indicate that serotonin and dopamine induce formation of H_2_S through upregulation and allosteric activation of CBS.

A fluorescent probe was used to assess the formation of ROS following rewarming of hypothermic cells (Figure 3F). Serotonin and dopamine did not affect ROS levels of control or CBS siRNA treated cells at 37°C. Hypothermic treatment of cells induced a strong increase in ROS formation, which was further aggravated in cells in which CBS was knocked out by siRNA. Serotonin and dopamine treatment completely normalized ROS production in cooled and rewarmed control cells. In CBS siRNA treated hypothermic cells, serotonin and dopamine only slightly reduced ROS production to a level observed in non-treated cells. Thus, CBS is necessary to convey the protective effect of serotonin and dopamine against the formation of ROS.

### Serotonin and dopamine upregulate CBS in cold-stored organs and attenuate apoptosis

**Figure 4:**
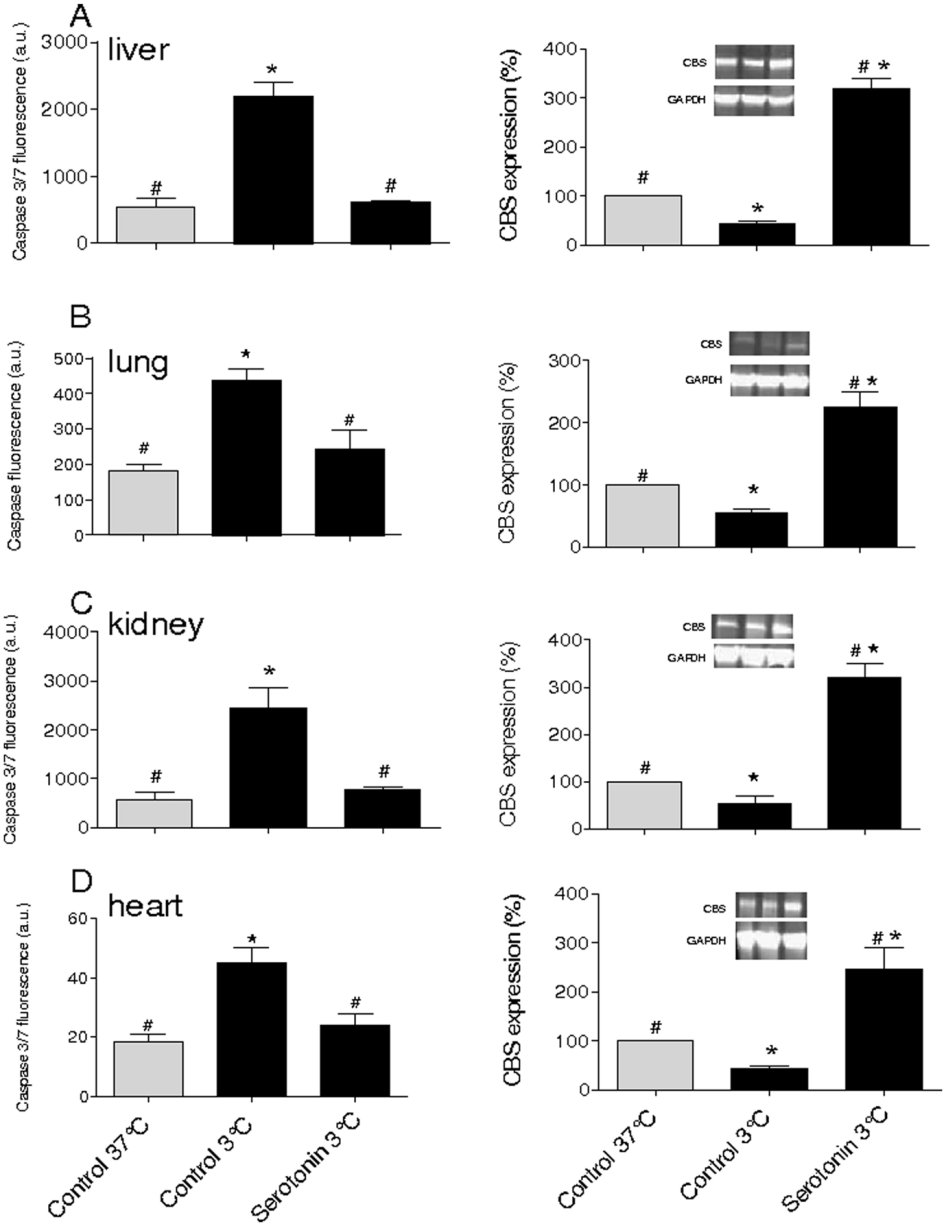
Serotonin increases cystathionine-β-synthase (CBS) expression and minimizes apoptosis in rat tissue during cold storage. Rat tissues were cut in slices in isotonic PBS and subjected to 24 h storage at 3°C followed by processing for analysis by fixation or snap-freezing. (**A** to **D**) cold storage of indicated tissues activates caspase 3/7 (left panels) and downregulates CBS (right panels), which is reversed by serotonin pretreatment (90 µM, 30 min) of tissues. Insets show typical examples of western blots. Western blot: left lanes: fresh tissue (Control 37°C), middle lanes: tissue stored for 24 h at 3°C (Control 3°C), right lanes: tissue pretreated with dopamine prior to storage at 3°C (Dopamine 3°C). ANOVA tests, different from non-cooled cells (Controls 37°C) P<0.05 (*); different from untreated hypothermic cells (Controls 3°C) P<0.05 (#). Experiments consist of n≥3. Means ± SEM.

**Figure 5:**
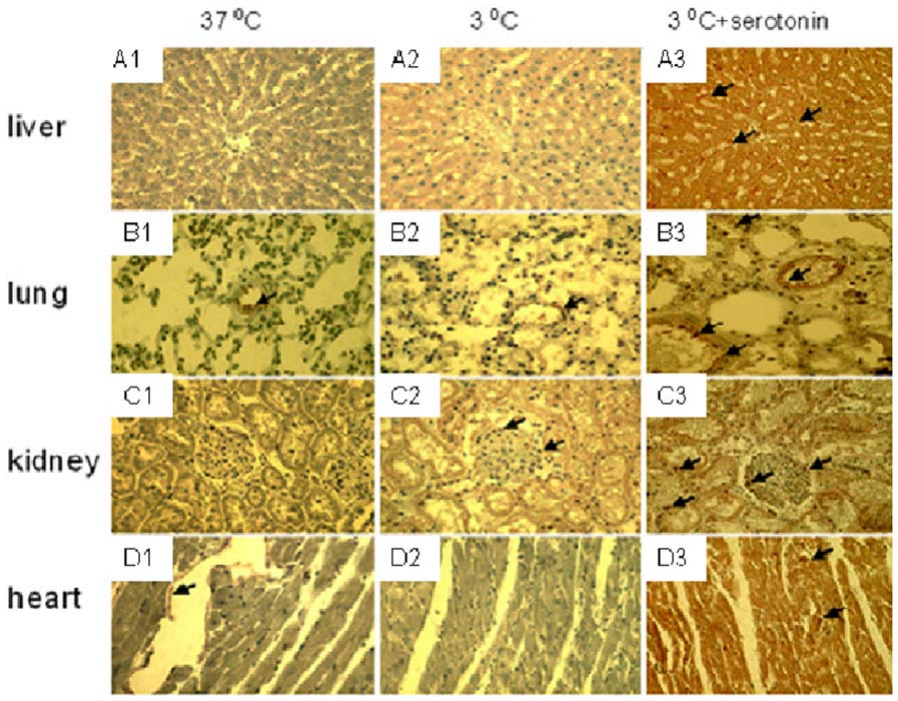
Localization of serotonin induced increase in cystathionine-β-synthase (CBS) expression during cold storage. Preincubation of slices with serotonin (90 µM, 30 min) and the subsequent 24 hr of hypothermic storage (3°C) causes substantial increase in the expression of CBS (A3-D3) compared to freshly processed control tissue (37°C, A1-D1) or nontreated cooled controls (3°C, A2-D2). Rat tissues were cut in slices in isotonic PBS and subjected to 24 h at 3°C, followed by processing for analysis by fixation. CBS is represented by brown staining and indicated by arrows. Magnification 40×.

Finally, to examine whether serotonin and dopamine induce the upregulation of CBS in organs as seen in cells, rat heart, liver, kidney, and lung were pre-incubated with serotonin (90 µM) and dopamine (60 µM) for 30 min prior to cold exposure (3°C, 24 h) and fixed 30 min after rewarming. In serotonin treated liver, lung, kidney and heart, CBS expression is higher following the hypothermic treatment and rewarming as compared to controls (Figures 4 and 5). Furthermore, serotonin pretreatment prevented activation of caspase in these tissues following prolonged cold storage and subsequent rewarming (Figure 4). Similar effects were observed in organs treated by dopamine (data not shown).

To assess tissue hypoxia after hypothermic preservation, the pH of the preservation medium was measured after rewarming. Storage and rewarming induced substantial acidosis in untreated tissues. Pretreatment with dopamine and serotonin prevented acidosis and maintained the physiological pH value with significant differences compared to the untreated tissues which suffered acidosis (Table S1).

## Discussion

Our data show that the cellular uptake of serotonin and dopamine prevents hypothermia/ rewarming induced cell apoptosis by H_2_S formation through CBS upregulation and probably allosteric activation. Both compounds attenuate the increase in ROS formation in cells subjected to hypothermia/rewarming. The ROS inhibitory action of dopamine or serotonin in cooled SMAC was minimized after siRNA mediated knock-down of CBS protein. Moreover the attenuation of CBS upregulation by rapamycin treatment of SMAC, points at a potential beneficial effect of mTOR activation in hypothermia/rewarming induced damage through upregulation of CBS and production of H_2_S. In accord, PLP as an activator of CBS and NaHS as a substance which releases H_2_S also protected against cell death induced by hypothermia/rewarming. Finally, whereas cooling downregulates CBS in various rat tissues as observed in our experiments, dopamine and serotonin attenuate and even upregulate CBS expression throughout the treatment and protect against acidosis and apoptosis. Thus, we expand the previous findings on cell protective properties of dopamine and serotonin [6], [19] and identify the activation of H_2_S pathway as a main effector in prolonged protection against hypothermia/rewarming damage.

Previous data corroborate the presence of serotonin filled vesicles in vas deferens smooth muscle from which DDT-1 cells are derived. Fuenmayor et al. [20] and Celuch and Sloley [21] described the presence and release of serotonin, dopamine and noradrenalin (NA) from rat vas deferens. It is conceivable that protection from hypothermia in SMAC cells is dependent on the cellular uptake of serotonin, in view of the failure of its protection in the presence of an SSRI and the unchanged effectiveness of serotonin in the presence of the non-selective 5-HT_2_ receptor blocker ketanserin.

H_2_S is already known as being a cell and tissue protective molecule in ischemia/reperfusion damage. This study discloses the feasibility to limit hypothermia/ rewarming cell injury by increasing the endogenous production of H_2_S. In addition, it discloses a novel molecular link between two important biogenic amines and the H_2_S pathway. The protection by H_2_S against apoptotic injury and cell death following cooling and rewarming extends previous reports showing H_2_S to protect from hypoxic injury in cells and tissues, and in animals [9], [22]. However, it should also be noted that a number of studies identified a lack of effect of H_2_S administration, mainly in larger animals such as sheep or pig [23], [24]. Presently, the reason for the lack of protection by H_2_S administration under these conditions is unclear, but this may relate to kinetics of the H_2_S, particularly following bolus injection of H_2_S donors [25]. As our data show that the therapeutic potential of endogenously produced H_2_S may be disclosed via a relatively simple pharmacological approach to protect against cold ischemia-reperfusion injury and it will be of interest to explore the effectiveness of compounds boosting endogenous H_2_S production in organ transplantation models in larger animals. This is the first report of serotonin and dopamine limiting cellular damage following cooling and rewarming through H_2_S production. The mechanism by which H_2_S attenuates apoptosis is unknown, but has already been suggested to constitute of compensation for the loss of SH-reduction equivalents during cold preservation [26], or alternative mechanisms [27], [28]. On the molecular level, various signal transduction pathways downstream of H_2_S have been implicated (reviewed in [9]), including the opening of ATP-sensitive K^+^ channels, activation of eNOS and the activation of pro-survival kinases ERK, PKC isoforms and PI3K-Akt, resulting in augmented expression of heat shock proteins, Bcl-2 and Bcl-xL. Dopamine has been shown previously to protect from hypothermia induced apoptosis in cultured cells [6], [26] and to improve graft patency in human kidney transplantation [7]. This beneficial effect has been mainly contributed to its antioxidant properties [26]. As its already known that dimerization and reduction of serotonin and dopamine in the presence of free radicals occurs very quickly annihilating the antioxidant effect [6], [29] our results on the production of H_2_S through CBS following dopamine and serotonin treatment may further expand and fortify the protective effect of these compounds. The importance of CBS in redox regulation and reaction mechanism has already been reviewed [30] although a direct link to cellular resistance against ROS due to the presence of CBS has never been made. Further, as a contradictive addition to the above report on dopamine and serotonin being antioxidants, these bioamines have also the ability to induce ROS formation in cells, for example due to the activation of proteins in mitochondrial metabolic pathways such as monoamine oxidases (MAOs) [31], [32]. ROS formation was not observed in our cells indicating a potential inhibition of MAOs, which could also be contributed to the possible inhibition of these enzymes by H_2_S [33].

Our study demonstrates that the increased H_2_S production in cells is due to both upregulation and allosteric activation of CBS. The upregulation of CBS expression following serotonin and dopamine incubation is most likely caused via activation of mTOR kinase and subsequent activation of the protein synthesis machinery. In accord, serotonin and dopamine have been shown to activate mTOR through their respective receptors [34] in this case indicating the beneficial role of this kinase in protection against hypothermia/rewarming damage and ROS formation through CBS upregulation.

A second way of enhancing H_2_S production by serotonin and dopamine constitutes of the allosteric activation of CBS. The exact nature of the interaction of serotonin and dopamine with CBS needs further exploration. However, as biogenic amines were previously found to modulate the catalytic activity of various heme enzymes [12], the N-terminal heme moiety of CBS constitutes an important candidate as the site of interaction. In addition, CBS is known to be allosterically modulated by S-adenosyl-L-methionine (SAM) via interaction with its C-terminal (regulatory) domain, increasing its activity about 3-fold [35]. Our finding that dopamine and serotonin upregulate CBS in four different cold stored organs, even in the ones such as lung and heart which were previously identified to not exhibit CBS activity in rats [36], hints at a presence and importance of the system in different organs. Although CBS enzyme activity is not found in all cells, its expression in brain, liver and kidney is known to be substantial [37]. The profound CBS upregulation found in cold stored liver in the present study is in agreement with the high expression of CBS reported in this organ [22], [37]. One of the consequences of hypothermia is cold induced hypoxia [38], which in turn induces tissue acidosis [39]. In mammalian cells, regulation of basic cell membrane function is closely linked to cellular pH and a stable tissue pH is considered to reflect cell viability [40]. The assessment of pH value of the tissue medium after hypothermic preservation demonstrates tissue acidosis in control tissues and its absence in treated tissues, suggesting maintenance of membrane integrity in cold induced acidosis of tissue probably due to a lower ROS production. The importance of CBS in peripheral organs is underscored by the phenotype of genetic defects of the CBS gene. In humans, genetic mutations invoking CBS deficiency lead to the clinical condition of homocystinuria, not only characterized by severe disorders of brain, but also of eyes, and the musculoskeletal and cardiovascular system [41]. Also in heterozygote CBS knock-out mice, abnormalities in liver [41], kidney [42] and the cardiovascular [43] and pulmonary system [44] are prominent. Together, these expression profiles indicate crucial roles for CBS activity.

In broader sense, our results identified a novel molecular link between major monoamine neurotransmitters and the H_2_S pathway. While prominently expressed in brain, a significant expression of the uptake pumps for serotonin and dopamine, SERT and DAT, was also found in peripheral organs, e.g. in liver, kidney and lung [45], [46]. Various drug classes profoundly affect serotoninergic and dopaminergic systems, including medicines such as anti-depressants (reviewed in [47]) but also recreational drugs including cocaine, amphetamines and XTC. In theory, any drug that interferes with synthesis, cellular uptake and/or metabolism of these neurotransmitters may affect H_2_S signaling. In turn, H_2_S has been implicated in various physiological processes including modulation of blood pressure [48], [49] and neuromodulatory effects, including nociception [22], [50], [51]. To what extent CBS function and H_2_S production influence the physiological action of monoaminergic transmitters and the pharmacological effects of related drugs needs further exploration. Our results implicate that cells that do not express CBS are prone to oxidative injury even in the presence of serotonin and dopamine. Possibly, differences in CBS expression in different cells in an organ may explain conflicting data on the effects of these bioamines in various settings.

In summary, this study reveals that the cellular uptake of serotonin and dopamine limits cold induced cellular damage, ROS production, and apoptosis by CBS induced H_2_S formation. This finding discloses an additional effector pathway of biogenic amines and enlightens the potential of the CBS enzyme in attenuating oxidative stress.

## Supporting Information

Figure S1 **Natural resistance of DDT-1 cells to hypothermic damage is due to secretion of a hypothermia-protecting factor into medium of cooled cells.** Cells subjected to hypothermia (black bars) were incubated at 3°C for 24 h, followed by rewarming to 37°C for 3 h. Cell viability was assessed by adding MTS to the cells upon rewarming and spectrophotometrical formozan measurement. (**A**) DDT-1 cells show natural resistance to hypothermia/rewarming, in contrast to THMC (transformed human mesangial cell), A7R5 (rat vascular smooth muscle cells), SMAC (rat smooth muscle aortic cells) and NRK (normal rat kidney cells). (**B**) Hypothermia/rewarming injury of vulnerable cell lines is precluded when the protocol is executed in medium from cooled DDT-1 cells (conditioned medium from 3°C cells: CM 3°C), whereas medium from non-cooled DDT-1 cells (CM 37°C) is not protective. ANOVA tests, different from non-cooled cells (37°C ). P<0.05 (*); different from CM 37°C conditioned cells P<0.05 (#).Experiments consist of n≥3. Means ± SEM.
(TIF)

Figure S2 **DDT-1 cells contain serotonin filled vesicles.** (A and B) show representative photographs of DDT-1 cells stained with Ehrlich reagent (A; blue color) and serotonin antibody (B; brown color), respectively.
(TIF)

Figure S3 **Upregulation of cystathionine-β-synthase (CBS) expression by dopamine and H_2_S production by isolated enzyme.** **A.** Treatment with dopamine (20 µM, 15 min at 37°C+24 hr at 3°C) upregulates CBS expression in SMAC cells, which is inhibited by pretreatment with rapamycin (rap, 30 nM). Inset: typical western blot with time points as indicated. ANOVA tests, different from non-treated cells (0) P<0.05 (*).Experiments consist of n≥3. Means ± SEM. **B.** Serotonin and dopamine induce H_2_S production by CBS *in vitro* at 37°C, as does the endogenous activator of CBS, pyridoxal 5-phosphate (PLP) ANOVA tests, different from non-cooled cells (37°C or Con ) P<0.05 (*); different from untreated hypothermic cells (Con) P<0.05 (#); different from min serotonin treated cells P<0.05 (&). Two way ANOVA with Bonferroni, different from substrate incubated cells P<0.01 (‡). Experiments consist of n≥4. Means ± SEM.
(TIF)

Table S1
**pH values of medium of tissue slices following rewarming.** Preincubation of slices in 2 ml of PBS containing serotonin (90 µM), dopamine (60 µM) or PBS with no treatment (vehicle) for 30 min followed by 24 hr of hypothermic storage (3°C) and 30 min of rewarming (37°C) causes acidosis in medium of control tissues compared to those tissues treated with serotonin and dopamine. The data each represent the mean of 3 separate experiments (Mean
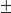
SEM) * significantly different compared to vehicle treated controls within each tissue group.(DOC)

Text S1 **Caspase activity measurement in cells and tissue samples using Promega Apo-ONER assay obtaining conditioned medium from DDT-1 cells by cold storage.** Inhibition of serotonin synthesis by parachlorophenyl-alanine (PCPA) incubation. Quantitative assessment of serotonin in cells by Ehrlich's reagent and mass spectrometry. Western Blot conditions and detection of protein bands in samples from cells and tissue. Histology and immunostaining procedures in cells and tissue slices. Measurement of reactive oxygen species.
(DOC)
