# Supplementary figures and images for: Serotonin and Dopamine Protect from Hypothermia/Rewarming Damage through the CBS/ H2S Pathway
Source: PLoS One. 2011 Jul 27;6(7):e22568. doi: 10.1371/journal.pone.0022568 (PMC3144905; doi:10.1371/journal.pone.0022568)

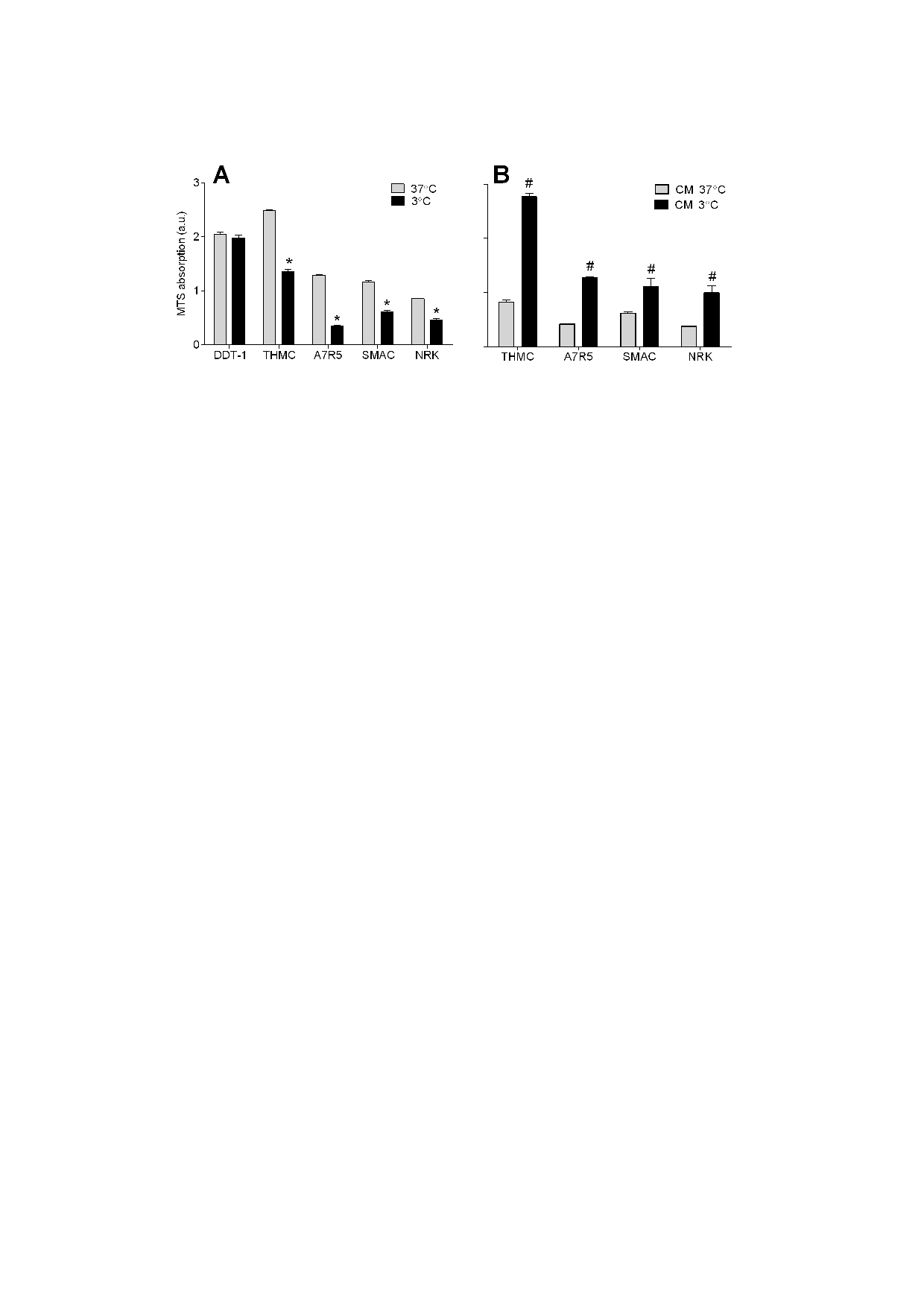

Supplement: Figure S1 — Natural resistance of DDT-1 cells to hypothermic damage is due to secretion of a hypothermia-protecting factor into medium of cooled cells. Cells subjected to hypothermia (black bars) were incubated at 3°C for 24 h, followed by rewarming to 37°C for 3 h. Cell viability was assessed by adding MTS to the cells upon rewarming and spectrophotometrical formozan measurement. (A) DDT-1 cells show natural resistance to hypothermia/rewarming, in contrast to THMC (transformed human mesangial cell), A7R5 (rat vascular smooth muscle cells), SMAC (rat smooth muscle aortic cells) and NRK (normal rat kidney cells). (B) Hypothermia/rewarming injury of vulnerable cell lines is precluded when the protocol is executed in medium from cooled DDT-1 cells (conditioned medium from 3°C cells: CM 3°C), whereas medium from non-cooled DDT-1 cells (CM 37°C) is not protective. ANOVA tests, different from non-cooled cells (37°C ). P<0.05 (*); different from CM 37°C conditioned cells P<0.05 (#).Experiments consist of n≥3. Means ± SEM. (TIF) [file pone.0022568.s001.tif]

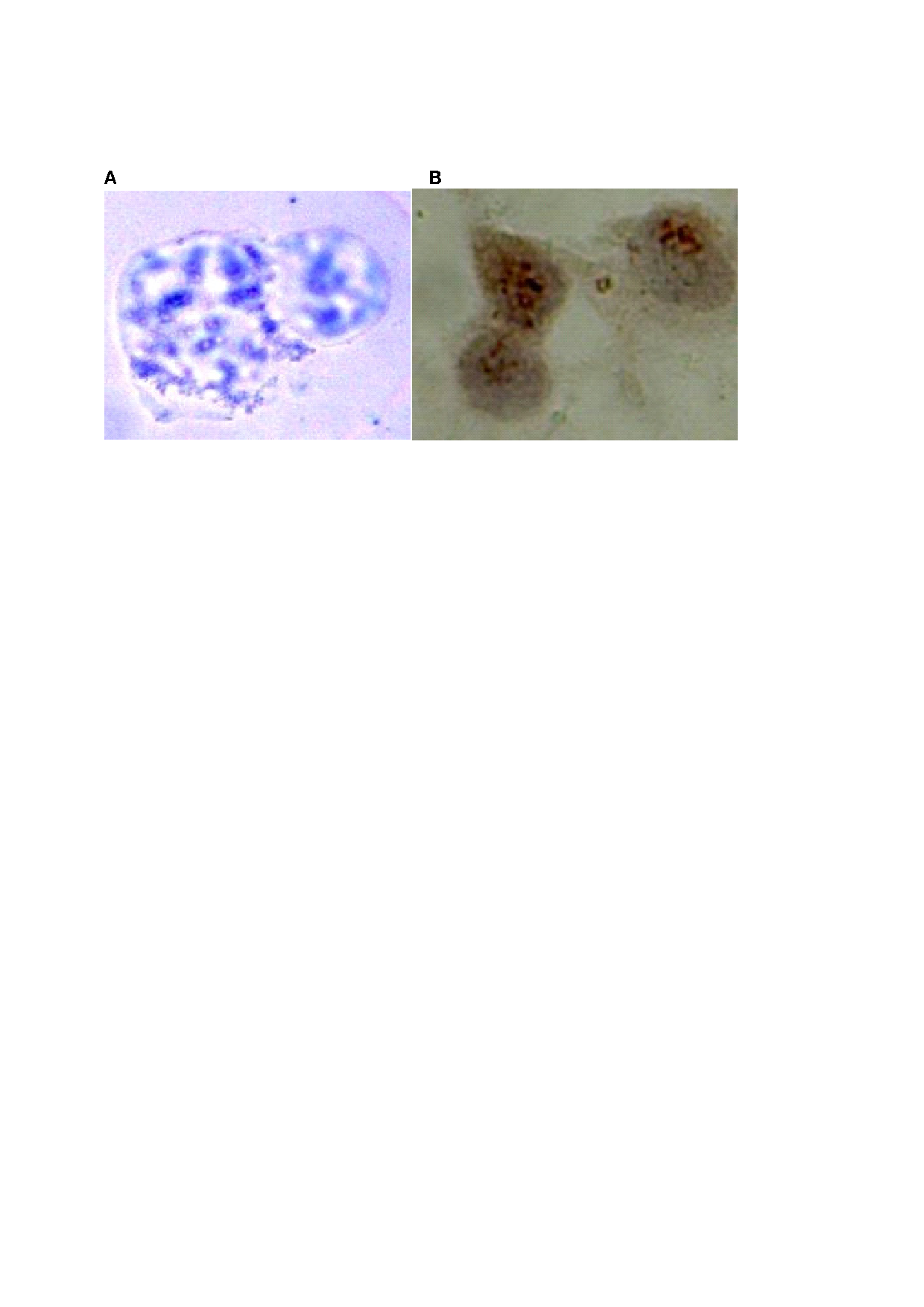

Supplement: Figure S2 — DDT-1 cells contain serotonin filled vesicles. (A and B) show representative photographs of DDT-1 cells stained with Ehrlich reagent (A; blue color) and serotonin antibody (B; brown color), respectively. (TIF) [file pone.0022568.s002.tif]

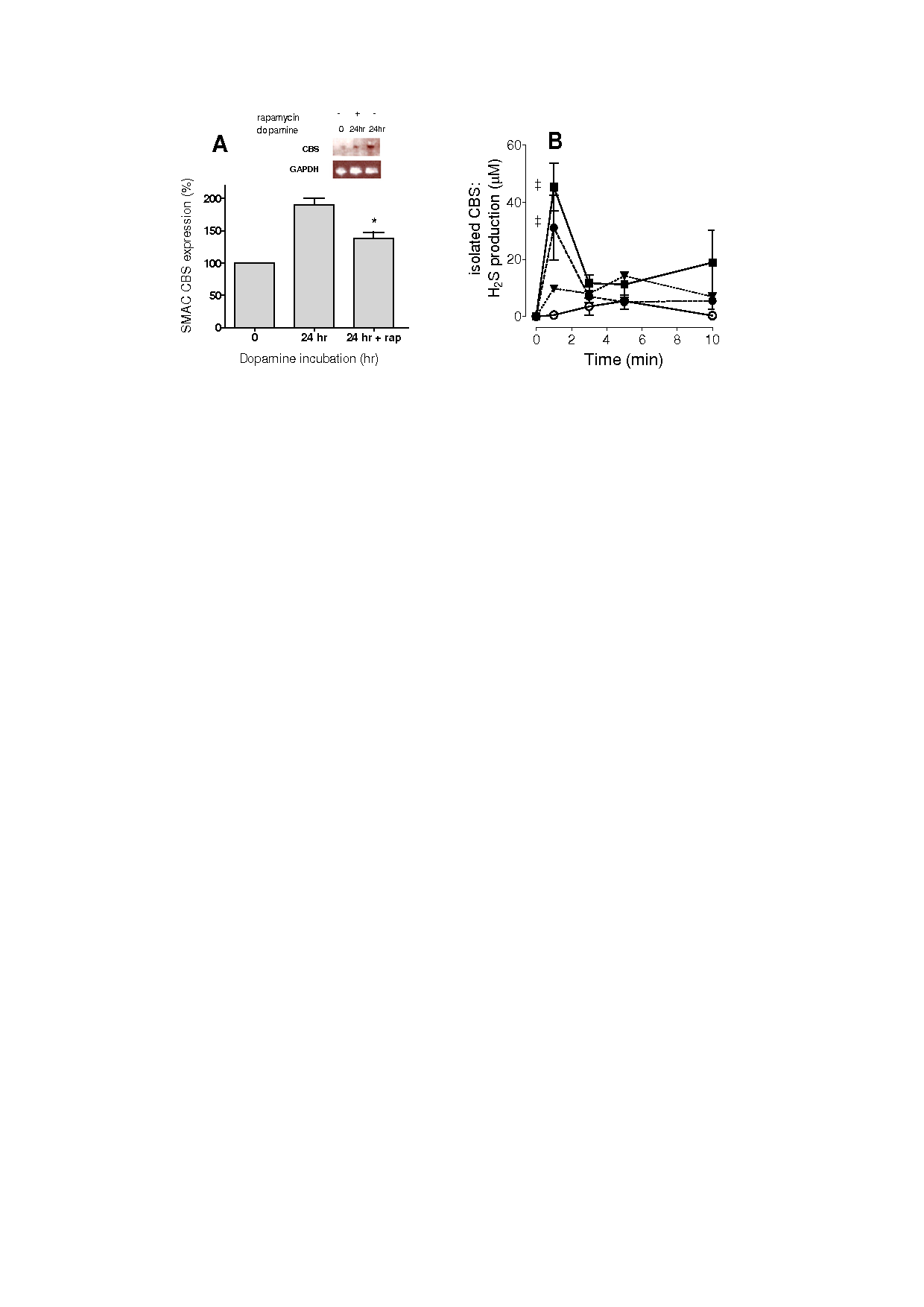

Supplement: Figure S3 — Upregulation of cystathionine-β-synthase (CBS) expression by dopamine and H2S production by isolated enzyme. A. Treatment with dopamine (20 µM, 15 min at 37°C+24 hr at 3°C) upregulates CBS expression in SMAC cells, which is inhibited by pretreatment with rapamycin (rap, 30 nM). Inset: typical western blot with time points as indicated. ANOVA tests, different from non-treated cells (0) P<0.05 (*).Experiments consist of n≥3. Means ± SEM. B. Serotonin and dopamine induce H2S production by CBS in vitro at 37°C, as does the endogenous activator of CBS, pyridoxal 5-phosphate (PLP) ANOVA tests, different from non-cooled cells (37°C or Con ) P<0.05 (*); different from untreated hypothermic cells (Con) P<0.05 (#); different from min serotonin treated cells P<0.05 (&). Two way ANOVA with Bonferroni, different from substrate incubated cells P<0.01 (‡). Experiments consist of n≥4. Means ± SEM. (TIF) [file pone.0022568.s003.tif]
