# Supplementary material for: Serotonin and Dopamine Protect from Hypothermia/Rewarming Damage through the CBS/ H2S Pathway
Source: PLoS One. 2011 Jul 27;6(7):e22568. doi: 10.1371/journal.pone.0022568 (PMC3144905; doi:10.1371/journal.pone.0022568)
Supplement: Table S1 — pH values of medium of tissue slices following rewarming. Preincubation of slices in 2 ml of PBS containing serotonin (90 µM), dopamine (60 µM) or PBS with no treatment (vehicle) for 30 min followed by 24 hr of hypothermic storage (3°C) and 30 min of rewarming (37°C) causes acidosis in medium of control tissues compared to those tissues treated with serotonin and dopamine. The data each represent the mean of 3 separate experiments (MeanSEM) * significantly different compared to vehicle treated controls within each tissue group. (DOC) [file pone.0022568.s004.doc]

| Tissue  Treatment | Liver | Heart | Kidney | Lung |
| --- | --- | --- | --- | --- |
| Vehicle | 6.9±0.1 | 6.8±0.2 | 6.8±0.2 | 6.8±0.1 |
| Dopamine | 7.4±0.1* | 7.3±0.1* | 7.3±0.1* | 7.3±0.1* |
| Serotonin | 7.3±0.1* | 7.3±0.2* | 7.3±0.2* | 7.3±0.2* |

**Table. S1.** **pH values of medium of tissue slices following rewarming.** Preincubation of slices in 2 ml of PBS containing serotonin (90 µM), dopamine (60 µM) or PBS with no treatment (vehicle) for 30 min followed by 24 hr of hypothermic storage (3ºC) and 30 min of rewarming (37ºC) causes acidosis in medium of control tissues compared to those tissues treated with serotonin and dopamine. The data each represent the mean of 3 separate experiments (Mean$\pm$SEM) * significantly different compared to vehicle treated controls within each tissue group.
