# Supplementary material for: Serotonin and Dopamine Protect from Hypothermia/Rewarming Damage through the CBS/ H2S Pathway
Source: PLoS One. 2011 Jul 27;6(7):e22568. doi: 10.1371/journal.pone.0022568 (PMC3144905; doi:10.1371/journal.pone.0022568)
Supplement: Text S1 — Caspase activity measurement in cells and tissue samples using Promega Apo-ONER assay obtaining conditioned medium from DDT-1 cells by cold storage. Inhibition of serotonin synthesis by parachlorophenyl-alanine (PCPA) incubation. Quantitative assessment of serotonin in cells by Ehrlich's reagent and mass spectrometry. Western Blot conditions and detection of protein bands in samples from cells and tissue. Histology and immunostaining procedures in cells and tissue slices. Measurement of reactive oxygen species. (DOC) [file pone.0022568.s005.doc]

**Caspase Activity Measurement.** Tissue samples were lysed in 50 mM potassium phosphate buffer (pH 6.9, 5%, w/v), sodium orthovanadate 10 mM and protease inhibitor. Bradford assay was conducted and 500 µg of protein was deposited in each well of a black Immuno plate (Nunc) and diluted to 50 µl with the lysis buffer. Fifty µl of caspase substrate (Promega Apo-ONE®) was added to each well. The plates were left on a shaker for 3 h and fluorescence was read at an excitation of 499 nm and emission maximum at 521 nm. For measurement of caspase activity in cells, medium was removed and replaced by 50 µl of new medium and left for 3 h. Fifty µl of caspase substrate was added to each well and measured as described above.

**Conditioned Medium.** DDT-1 cells were grown to confluence in 25 cm^2^ flasks, washed with PBS, incubated with 5 ml of fresh medium and placed at 3 ºC or 37 ºC for 18 h to obtain conditioned mediums (CM3 and CM37, respectively). CM was filtered through a 0.2 μm cellulose acetate disposable filter unit (Whatman) and stored at -20 ºC until use. NRK, SMAC, A7R5 and THMC cells were grown to confluence in 96 well plates. Thereupon, the supernatant was replaced by 200 μl of CM3 or CM37 and incubated at 37ºC for 15 minutes and subsequently placed at 3°C or 37°C for 24 h. Cell viability was assessed by MTS assay.

**Inhibition of Serotonin Synthesis.** Parachlorophenyl-alanine (PCPA; Sigma) was dissolved in warmed, acidified (pH 6.8) DDT-1 medium and vortexed for 5 min to a stock concentration of 1.25 μM. Control experiments were performed with a similar solution without PCPA. Creatine anhydrous (Sigma) was dissolved in cell medium and added to wells to exclude the effect of this component. The treatment continued for 4 days until the concentration of indoleamines inside the cells reached half of the initial value. The cells were subjected to hypothermia at 3ºC and MTS assay was performed after 24 h.

**Quantitative Assessment of Serotonin and indoles in Cells.** Ehrlich’s reagent was used to quantify the cellular amount of indoles. Qualitative analysis of cellular indoles in cell culture medium at 37º C and 3º C was conducted after extraction according to Happold and Hoyle [1]. Five ml of medium was shaken vigorously with 2 ml of xylene. Next, 1 ml of Ehrlich's reagent was applied to the surface of the mixture. Redistribution of xylene through the Ehrlich's reagent induces formation of the rosindole body, a red ring appearing at the lower surface of the xylene layer indicating the presence of indoleamides. The change in indole concentration in DDT-1 cells was measured after washing the cells with PBS, centrifugation (1000 rpm, 5 min) and removal of supernatant. Ehrlich reagent (200 μl) was added to each tube. After 3 min of vortexing, tubes were left for 3 h at 60 ºC. After centrifugation (1000 rpm, 5 min), color intensity was spectrophotometrically measured at 625 nm. Calibration experiments were carried out using serotonin (0.025- 0.5 mM), which rendered a linear regression with a correlation coefficient (R^2^) of 0.9996 (p<0.01; n=3). To verify the accuracy of the Ehrlich reagent experiments automated mass spectrometric analysis was performed on all the samples according to the method of de Jong *et al.*[2].

**Western Blot.** Treated cells (SMAC) in 6 well plates were washed with PBS and lysed in 120μl RIPA buffer (1 mM EDTA, 1% Triton X-100, 0.1% sodium deoxycholate, 0.1% SDS, 140 mM NaCl) supplemented with protease inhibitor. The protein concentration was measured by Bradford assay in all the samples. Loading buffer (20 μl) was added to every 50 μg of cell protein and ran at 100V for 70 min. Proteins were transferred to a nitrocellulose membrane and incubated with antibodies as indicated. Expression was detected by West Pico Chemiluminescent Substrate (SuperSignal, Thermo Scientific), photographed and analyzed with genetool software (version 3.08, SynGene). Treated and untreated tissue sections were snap frozen in liquid nitrogen and homogenized in dry ice and RIPA lysing buffer. The protein concentration in the sample tissues were measured according to Bradford protein assay. 50 μg of lung samples mixed with loading buffer were boiled and loaded onto 4-20% Precise™ protein gel (Thermo Scientific). The proteins were transferred onto a nitro-cellulose membrane and probed by CBS antibody (1:500) and a 1% secondary antibody Goat AntiRabbit HRP (Dako, po448). The membranes were developed using supersignal West Dura substrate and syngene version 6.07 was used to capture the illuminated bands representing the level of protein expression. The band intensities obtained from CBS protein were corrected over GAPDH as an internal reference.

**Histology and Immunostaining.** For histological examination serotonin, cells were fixed by acetone and stained using two methods. Ehrlich reagent was used after fixation with acetone (100%) for 10 min. Ehrlich’s reagent was prepared by dissolving 100 mg p-dimethylaminobenzaldehyde in 100 ml 17:3 (v/v) glacial acetic acid / hydrochloric acid mixture and stored at 4 ^0^C until later use. Fixated cells were placed inside a glass chamber containing 2% Ehrlich reagent and heated at 60ºC for 30 min. Next, slices were washed with PBS and examined using a light microscope. For immunohistological examination, cells were fixed by acetone (100%) for 10 min, washed and rehydrated with PBS. Hydrogen peroxidase activity was blocked by hydrogen peroxide (1%) in PBS, washed with PBS three times for 5 min each and incubated for 1 h with 1% primary antibody to serotonin (Abcam, ab8882-50) in PBS containing 1% BSA for 1 h, washed in PBS thrice and incubated with 1% secondary antibody Goat AntiRabbit HRP (Dako, po448) in PBS containing 1% BSA for 1 h and again washed in PBS thrice. The signal was amplified by a 1% of a third antibody, rabbit Anti Goat (Dako, po449) in medium. For immunohistological examination of CBS protein the same procedure was followed to prepare the cells. Fixed cells were covered by the first antibody, CBS antibody (Santa Cruz, sc-46830), in PBS containing 1% BSA. A similar procedure with secondary and tertiary antibodies was followed as described above. A similar procedure was followed to stain CBS in rat tissue slices. Dako AEC+ High Sensitivity Substrate Chromogen (k3469) was used in all staining to visualize protein bound antibody. 10 min Haematoxylin counterstaining visualized the nuclei.

**Measurement of reactive oxygen species.** SMAC were cultured in two 96-well black Immuno plate (Nunc). Two rows in each plate was treated with CBS siRNA and left for 24 hr at 37ºC to reach confluence. Later the cells were treated with either serotonin (30 µM) or dopamine (20 µM), keeping the nontreated rows as controls. After 15 min the plates were placed at 3ºC for 24 hr. Later the plates were placed inside the incubator (37ºC, 30 min). Normothermic controls (37ºC) were processed in the same order excluding the hypothermia preservation stage. The medium in each well was then emptied and 20 µl of Ripa buffer was added to each well to homogenize cells, plates were then left for 10 min on a shaker. Oxygen radical formation was determined by loading homogenates in 40mM Tris, pH 7.4 with CM-H2-DCFDA in methanol for 15 min. Serial measurement in a Fluorescent 96-well plate reader was performed. The formation of the fluorescent probe DCF (Polysciences Inc., Warrington, PA) was monitored at excitation wavelength 488 nm (bandwidth 5nm), and emission wavelength 525 nm (bandwidth 20 nm). The cuvette holder was thermostatically maintained at 37°C. Auto fluorescence was subtracted prior to DCF fluorescence.

References

1. Happold FC, Hoyle L. (1934) The quantitative determination of indole in bacterial cultures. Biochem J 28: 1171-1173.

2. de Jong WH, Wilkens MH, de Vries EG, Kema IP. (2010) Automated mass spectrometric analysis of urinary and plasma serotonin. Anal.Bioanal.Chem. 396: 2609-2616.
